# Building Blocks of Self-Sustained Activity in a Simple Deterministic Model of Excitable Neural Networks

**DOI:** 10.3389/fncom.2012.00050

**Published:** 2012-08-06

**Authors:** Guadalupe C. Garcia, Annick Lesne, Marc-Thorsten Hütt, Claus C. Hilgetag

**Affiliations:** ^1^School of Engineering and Science, Jacobs University BremenBremen, Germany; ^2^Institut des Hautes Études ScientifiquesBures-sur-Yvette, France; ^3^Laboratoire de Physique Théorique de la Matière Condensée, UMR 7600, Université Pierre et Marie Curie-Paris 6Paris, France; ^4^University Medical Center Eppendorf, Hamburg UniversityHamburg, Germany; ^5^Department of Health Sciences, Boston UniversityBoston, MA, USA

**Keywords:** self-sustained activity, cycles, excitable dynamics, cellular automaton

## Abstract

Understanding the interplay of topology and dynamics of excitable neural networks is one of the major challenges in computational neuroscience. Here we employ a simple deterministic excitable model to explore how network-wide activation patterns are shaped by network architecture. Our observables are co-activation patterns, together with the average activity of the network and the periodicities in the excitation density. Our main results are: (1) the dependence of the correlation between the adjacency matrix and the instantaneous (zero time delay) co-activation matrix on global network features (clustering, modularity, scale-free degree distribution), (2) a correlation between the average activity and the amount of small cycles in the graph, and (3) a microscopic understanding of the contributions by 3-node and 4-node cycles to sustained activity.

## 1 Introduction

The fiber networks linking the neural nodes of the brain possess a specific, non-regular, and non-random organization. This network organization comprises characteristic topological features, such as network motifs (small sets of nodes with specific wiring; Milo et al., 2002, 2004; Sporns and Kötter, 2004; Song et al., 2005), modules (sets of nodes with more internal than external connections; Hilgetag et al., 2000), and hubs (network nodes with a much higher-than average number of connections; Sporns et al., 2007; Zamora-López et al., 2010). These features may be present across many orders of scale, from circuits and populations of individual neurons (Mountcastle, 1997; Binzegger et al., 2004) to large-scale regions and lobes of the entire brain (Bullmore and Sporns, 2009), creating an intricate multi-scale organization of structural brain networks.

What are the consequences of this characteristic neuroanatomical network organization for neural dynamics during spontaneous network activity or task-related stimulation? The global dynamics of the brain display a number of characteristic features. As a central aspect, the brain shows self-sustained, rhythmic multi-frequency activity in the absence of external stimuli. Such rhythmic sustained activity represents internal self-organized states of the nervous system, and has attracted great attention (van Vreeswijk and Sompolinsky, 1996, 1998; Brunel, 2000; Roxin et al., 2004; Galán, 2008). Self-sustained brain dynamics are important in various neural functions, such as dynamic stability (Kaiser et al., 2007a; Kaiser and Hilgetag, 2010), signal propagation (Vogels and Abbott, 2005), and neural coding (Lewis et al., 2009). It was shown that networks of sparsely connected spiking neurons can produce highly irregular chaotic activity without external stimuli, due to the balance between excitation and inhibition (van Vreeswijk and Sompolinsky, 1998; Vogels and Abbott, 2005). However, little is known about the impact of neural network topology on the organization of sustained rhythmic neural activity.

Traditional perspectives of cognitive neuroscience saw the brain as a passive, stimulus-driven device. In this view, the spontaneous ongoing activity of the brain was regarded as noise. Over the last decades, the paradigms have shifted to consider the brain as an active network that can generate meaningful activity by itself, which has significant impact on the selective responses to stimuli (Engel et al., 2001; Fries, 2005; Knight, 2007). Importantly, the sustained resting-state activity of the brain is far from simply noisy. Rather, rhythmic oscillations with characteristic frequencies, such as α, β, γ waves are abundant (Buzsáki, 2006). The relationship between cognitive functions and brain rhythms as well as synchronization of the rhythms has been a central topic in neuroscience over the last decade (Engel et al., 2001; Fries, 2005). How oscillations emerge in the brain and how they are related to the network architecture, however, is still largely an open question.

More generally, studying the interplay between the modular architecture and dynamical activity of neural networks may deliver important insights for the understanding of structure-function relationships in neural systems. Previous investigations have shown several interesting results. The study of synchronization dynamics in the cerebral cortical network demonstrated that modular functional networks coincide with the anatomical communities (Zhou et al., 2006, 2007; Honey et al., 2009). In a model of spreading neural activity, persistent and scalable network activation could be produced in hierarchical modular networks, but not in same-size random networks, implying that the hierarchical cluster architecture is a potential basis for the stable and diverse functional patterns in cortical networks (Kaiser et al., 2007a; Kaiser and Hilgetag, 2010). It was also shown that hierarchical modular networks satisfy constraints of stability under dynamical changes (Robinson et al., 2009).

In the present paper, we are guided by the following questions: What aspects of network topology explain principal dynamic features of the brain, such as sustained activity, patterns of periodic activity, as well as large-scale, network-wide patterns of average activity? In particular, topological “devices” at what scale (e.g., at the level of nodes, motifs, cycles, or even more global network features, such as modules, hubs, or the network’s degree distribution) are responsible for the particular dynamic patterns?

We are exploring a highly minimal, deterministic model (a three-state cellular automaton) of a neuron, in order to extract a few “stylized facts” (Buchanan, 2012) of how network topology shapes excitable dynamics. The model is the deterministic limit of the stochastic cellular automaton analyzed in previous publications (Müller-Linow et al., 2006, 2008; Hütt and Lesne, 2009; Hütt et al., 2012). The deterministic nature of the model and the fact that we set the refractory period to a single time step lead to a high average excitation density. Generally, this model is not meant as a realistic rendering of neuronal dynamics, but rather as a means of extracting particularly strong influences of the topology on the dynamics.

Previous theoretical findings on self-sustained activity and excitable dynamics either followed from mean-field approaches or numerical simulations. In several cases (e.g., Deco et al., 2009, 2011), noise was considered to be essential for sustained activity. The establishment of sustained activity presumably depends on the initial conditions and the way the system is prepared. It has been shown (Carvunis et al., 2006) that the excitable dynamics on a scale-free graph of three-state neuron model (*E*: excited, *S*: susceptible, and *R*: refractory) with recovery period one settles in a period-three regime. The origin of this behavior has been identified in the presence of 3-node triangles of *ESR* acting as pacemakers and imposing their period on the whole system. Roxin et al. (2004) has shown, for integrate-and-fire neurons, that a very low density of shortcuts was sufficient to generate persistent activity from a local stimulus through the re-injection of activity into previously excited domains. In a continuous setting Qian et al. (2010b) and Liao et al. (2011) demonstrated the existence of phase-advanced driving links, revealing possible self-organized structures supporting self-sustained oscillations. Another study (Qian et al., 2010a) analyzed diverse self-sustained oscillatory patterns and their mechanisms in small-world networks of excitable nodes, showing that spatiotemporal patterns are sensitive to long-range connection probability and coupling intensity. Further research (Kaiser and Hilgetag, 2010) explored how variation in the number of hierarchical levels and the number modules per level influenced the network dynamics and the occurrence of limited sustained activity (between the extremes of either quickly dying out or activating the whole network).

Many of these previous investigations were done with continuous, ODE-based models. We here explore the simplest possible deterministic discrete excitable neuron model, a three-state cellular automaton, on a graph, in order to understand the topological factors contributing to self-sustained activity. With our minimal neuron model we are able to enumerate all system states for small devices contributing to sustained activity and explore transitions between systems states, much in the same way as in the case of Random Boolean Networks (Kauffman, 1969).

It is intuitively clear, and supported by numerical evidence (Qian et al., 2010b; McGraw and Menzinger, 2011; Vladimirov et al., 2012) that cycles contribute to sustained activity. Less clear is which cycles are selected and how their embedding in the network affects the periodicities observed in the excitation density. Our discrete model facilitates addressing these questions.

Our principal motivation is that some deep relationships between network topology and excitable dynamics are far more pronounced for the stylized deterministic dynamics discussed here. We show that topological network features at different scales sculpt different characteristics of the network dynamics. On the one hand, small cycles are responsible for the sustained activity of the whole network. On the other hand, large-scale features (such as hubs or modules) shape the organization of co-activation patterns. We start from a macroscopic perspective, where we identify global network properties regulating the co-activations patterns. Then we move to a more microscopic view, where we explore cycles as the sources of sustained activity.

## 2 Materials and Methods

### 2.1 Dynamic model

We studied a minimal deterministic discrete excitable model for a network of interacting elements. The model consisted of three discrete states for each node (susceptible *S*, excited *E*, and refractory *R*), which were updated synchronously in discrete time steps according to a set of update rules allowing for signal propagation: (1) a susceptible node *S* became an excited node *E*, when a direct neighbor was in the excited state; (2) an excited node *E* entered the refractory state *R*; (3) a node regenerated (*R* → *S*) after *r* time steps. The parameter *r* is the deterministic refractory period of the system. For small *r*, the network dynamics can settle into an regular oscillatory behavior after a transient period. The set of nodes is thus partitioned into distinct groups of nodes, where two nodes are in the same group when they are simultaneously excited. In the numerical results presented here, which were obtained for networks of a small to moderate size (∼60 nodes), we restricted ourselves to *r* = 1.

### 2.2 Analyzed networks

To investigate the role of the topology on the dynamics in a deterministic model, we considered three different types of bidirectional benchmark graphs: random, scale-free, and random-modular networks. The random graph was the classical Erdős–Rényi (ER) model (Erdős and Rényi, 1960), the scale-free graph was the Barabási–Albert (BA; Barabási and Albert, 1999) model, and the random-modular graph was a composition of four small random (ER) graphs of identical size with few links among them. All networks were generated with the software package NetworkX (Hagberg et al., 2008). The artificial networks had 60 nodes, and the BA and the random-modular network possessed 800 links. In the case of the ER graphs, the number of links was varied between 300, 400, 600, and 800 links. We also applied our minimal dynamical model to the cortical network of the cat (Scannell et al., 1999) and the network of macaque visual cortex (Felleman and Van Essen, 1991). The cat cortical network is composed of 52 cortical areas and 820 links among them, while the macaque visual cortex is composed of 30 areas and 311 links. Both data sets are available as part of the Brain Connectivity Toolbox (https://sites.google.com/a/brain-connectivity-toolbox.net/bct/; Rubinov and Sporns, 2010).

The adjacency matrix of the biological networks was reordered according to the community structure to better represent this feature. We used two algorithms implemented in the Brain Connectivity Toolbox (Rubinov and Sporns, 2010), one to compute the community structure and the other to reorder nodes maximizing the number of links close to the diagonal.

### 2.3 Co-activation matrices

After appropriate initialization of the deterministic dynamical model, the network activity settles into a regular periodic behavior. Therefore, the nodes are divided into distinct groups; nodes are in the same dynamic group when they are simultaneously active. To analyze the pattern of joint excitations, we computed the number of simultaneous excitations for all pairs (*i*, *j*) of nodes, and then normalized this value by the minimum number of excitations of node *i* and *j*. The outcome matrix is the so-called co-activation matrix, a representation of the functional connectivity of the nodes.

We also analyzed the co-activation matrix with time delay 1, i.e., the patterns of excitations when node *i* is active at time *t* + 1 and node *j* at time *t*.

We simulated the dynamics for 500 different initial conditions, and computed the average co-activation matrix for each network. This average co-activation matrix was used for all subsequent analyses.

The initial conditions were randomly generated, with probability 0.1 to set a node into the excited state *E*, and the remaining nodes were equipartitioned into susceptible *S* and refractory *R* states.

The topology structure of a network is represented by the adjacency matrix *A*, where *A_ij_* = 1, if node *j* is connected to node *i*, and *A_ij_* = 0 otherwise. To compare the adjacency matrix with the co-activation matrix, we thresholded and binarized the co-activation matrix into ones (exceeding the threshold) and zeros. To monitor the total number of non-zero entries in the thresholded co-activation matrix, we calculated the connection density, the total number of ones divided by the maximal possible number (*N*^2^ − *N*, where *N* is the number of nodes).

We calculated the Pearson correlation coefficient between the adjacency and co-activation matrices for different values of the threshold (we excluded the diagonal elements of both matrices to avoid spurious variations of the correlation coefficient). We benchmarked the results against the average correlation coefficient of binary random sequences with the same matrix dimension. This procedure was followed for 1000 different sequences and used to calculate an average correlation coefficient.

### 2.4 Counting cycles: Exact result with finite (end-constrained) recursion

A necessary condition for sustained activity in our model is the existence of cycles of nodes, in which activity propagates unidirectionally. To count elementary cycles (i.e., cycles where no vertices appear more than once in the sequence) on graphs, we implemented the following algorithm.

For a network of *N* nodes, we denote *B*^(*n*)^ the *N* × *N* matrix such that $B_{ij}^{\left(n\right)}$ gives the number of *distinct* paths of *n* steps, visiting *n* − 1 nodes *pairwise distinct* and distinct from *i* and *j*. The diagonal element $B_{ii}^{\left(n\right)}$ gives the number of *distinct* and *elementary* cycles of *n* steps passing through *i*. “Elementary” means that the cycle does not break into two or more cycles. In contrast, $A_{ii}^{n}$ gives the overall number of closed paths of length *n* passing through *i*, distinct but possibly embedding smaller loops. Each of the elementary *n*-cycles contains *n* − 1 other nodes, hence it is encountered *n* times when one lets *i* vary, i.e., each cycle should contribute with a weight 1/*n*. Overall the total number *𝒞*^(*n*)^ of distinct elementary cycles of length *n* is given by (note that this formula holds also for directed graphs):

(1) $$C^{\left(n\right)}=\frac{1}{n}\;\overset{N}{\underset{i=1}{\sum }}\;B_{ii}^{\left(n\right)}=\frac{1}{n}\;\text{Tr}B^{\left(n\right)}$$

This formula actually counts oriented cycles distinguishing, e.g., 12341 and 14321. It thus overestimates the number of undirected cycles by a factor of 2 for *n* ≥ 4. The first step of the computation writes:

(2) $$B_{ii}^{\left(n\right)}=\overset{N}{\underset{k=1}{\sum }}\;B_{ik}^{\left(n-1\right)}A_{ki}$$

Namely, a cycle of length *n* starting from *i* is obtained by closing any path starting from *i* and visiting exactly *n* − 1 pairwise distinct nodes. There is no need to restrict the sum to *k* ≠ *i* since this case is automatically discarded due to the fact that *A_ii_* = 0. We set *B*^(1)^ = *A*, then *B*^(2)^ = *A*^2^. Note that $B_{ii}^{\left(2\right)}$ is arguably not a number of cycles, as it accounts for degenerate cycles with two identical edges *i* → *k* and *k* → *i*. This number $B_{ii}^{\left(2\right)}$ actually coincides with the degree κ*_i_* of node *i* (number of edges *i* → *k* out of *i*). Anyhow, the values of the diagonal components of *B*^(2)^ will appear to be of no consequence in the recursion. The computation of $B_{ii}^{\left(3\right)}$ is still straightforward, according to the above formula. Non-trivial counting starts for *n* = 4. Indeed, the computation of $B_{ik}^{\left(3\right)}$ for *k* ≠ *i* already encounters the difficulty that will be present in the next steps of the recursion: we *cannot* compute $B_{ik}^{\left(3\right)}$ as $\sum_{j\neq i}\;B_{ij}^{\left(2\right)}A_{jk}$ since $B_{ij}^{\left(2\right)}$ contains the contribution of the path *i* → *k* → *j*, which leads to a 2-cycle *k* → *j* → *k* when adding the final step *j* → *k*, with contribution *A_jk_*. Similarly, for *n* > 4, computing $B_{ik}^{\left(n-1\right)}$ within a recursive scheme cannot be done simply by extending all loop-free paths of length *n* = 2. The difficulty is the same as for *n* = 4: in extending by a step *j* → *k* (contribution *A_jk_*) a path of length *n* − 2 from *i* to *j*, we have to exclude paths passing through *k* in order to obtain a (*n* − 2)-path actually contributing to $B_{ik}^{\left(n-1\right)},$ that is, with no loop. One thus has to keep track of the nodes actually visited, or not visited, by the paths. Accordingly, the computation of *B*^(*n*−1)^ will necessarily involve auxiliary matrices.

We define recursively auxiliary matrices $D^{\left(q|a_{1},\ldots ,a_{z}\right)}$ where *q* is an index for the power, equivalently path length, whereas *a*_1_,…, *a_z_* are the indexes of *z* excluded nodes:

(3) $$D_{ik}^{\left(q|a_{1},\ldots ,a_{z}\right)}=\underset{x\neq a_{1},\ldots ,x\neq a_{z},x\neq i}{\sum }D_{ix}^{\left(q-1|a_{1},\ldots ,a_{z},k\right)}A_{xk}$$

$D_{ik}^{\left(q|a_{1},\ldots ,a_{z}\right)}$ counts the paths of length *q* from *i* to *k* which do not visit nodes *a*_1_,…, *a_z_*. The recursion starts with

(4) $$D_{ik}^{\left(2|a_{1},\ldots ,a_{z}\right)}=\underset{x\neq a_{1},\ldots ,x\neq a_{z}}{\sum }A_{ix}A_{xk}\;$$

For *q* > 2, we have moreover to make sure that counted paths contain no loop, hence the presence of the additional excluded node *k* in the auxiliary matrix $D^{\left(q-1|a_{1},\ldots ,a_{z},k\right)}$ involved in the recursion formula (3). This ensures that the added node *k* (step *x* → *k*, contribution *A_xk_*) has not yet been visited by the path of length *q* − 1. Also, the sum has to exclude the case *x* = *i*, in order to get a loop-free contribution to *D*^(*q*|.)^ (no embedded cycle *i* → *i*). In any cases, the sum is restricted to *x* ≠ *a*_1_,…, *x* ≠ *a*_z_ to ensure that the resulting paths do not pass through the excluded nodes *a*_1_,…, *a_z_*. The computation of $D^{\left(q|a_{1},\ldots ,a_{z}\right)}$ ultimately involves matrices of the form $D^{\left(2|a_{1},\ldots ,a_{z},a_{z+1},\ldots a_{z+q-2}\right)}$ (at the first step of the recursion, from *D*^(2|·)^ to *D*^(3|·)^). This shows that an unbounded recursion (in the limit as *N* → ∞) is not possible. Indeed, the construction has to take into account up to which cycle length *n* we want to go, so as to compute right from the beginning matrices $D^{\left(2|a_{1},\ldots ,a_{z+q-2}\right)}$ with the proper number *n* − 3 of excluded sites. More precisely, these auxiliary matrices are used in the formula for loop-free (*n* − 1)-paths:

(5) $$B_{ik}^{\left(n-1\right)}=\underset{y\neq i}{\sum }D_{iy}^{\left(n-2|k\right)}A_{yk}\;$$

where $D_{iy}^{\left(n-2|k\right)}$ is the number of paths of length *n* − 2 from *i* to *y* which do not pass through *k*. Ultimately, we start from matrices $D^{\left(2|a_{1},\ldots ,a_{n-3}\right)}.$

For *n* ≥ 4, *D*^(*n*−2|*j*)^ is constructed recursively from $D^{\left(2|a_{1},\ldots ,a_{n-4},j\right)}.$ We thus need to keep track of $D^{\left(2|a_{1},\ldots ,a_{n-3}\right)}$ to go up to $B_{ii}^{\left(n\right)},$ and also of $D^{\left(2|a_{1},\ldots ,a_{n-3}\right)}$ to get $B_{ii}^{\left(q\right)}$ with *q* < *n*. To compute for instance the number of cycles of length up to *n* = 5, we need to compute the auxiliary matrices $D^{\left(2|a_{1}\right)},$ $D^{\left(2|a_{1},a_{2}\right)},$ and $D^{\left(3|a_{1}\right)}.$ More generally, to compute the number of cycles of length up to *n*, we need to keep track of all matrices $D^{\left(2|a_{1},\ldots ,a_{z}\right)}$ with *z* ≤ *n* − 3.

## 3 Results

### 3.1 Macroscopic analysis

We generated co-activation matrices for different network types and studied how the different topologies affected statistical properties of the pairwise co-activation of nodes. We considered node co-activations in the regime of sustained activity, which arises from a combination of the initial conditions and the topology of the networks. In particular, some initial conditions only lead to transient activity, while others lead to sustained activation, see Section 3.3.

In the deterministic model for a finite system, the only possible type of sustained activity is periodic. As the configuration space is finite, the trajectory of the system necessarily visits some configuration twice, where it will either stay (fixed point) or from which it will indefinitely follow the same periodic orbit (sustained activity) due to the deterministic nature of the dynamics. A non-trivial question is how much the topology of the network contributes to the successful implementation of deterministic sustained dynamics. In particular, it is not clear whether the pool of successful distributions of states systematically increases or decreases with the modification of certain topological features.

#### 3.1.1 Functional connectivity indicated by node co-activation patterns

**Figure 1:**
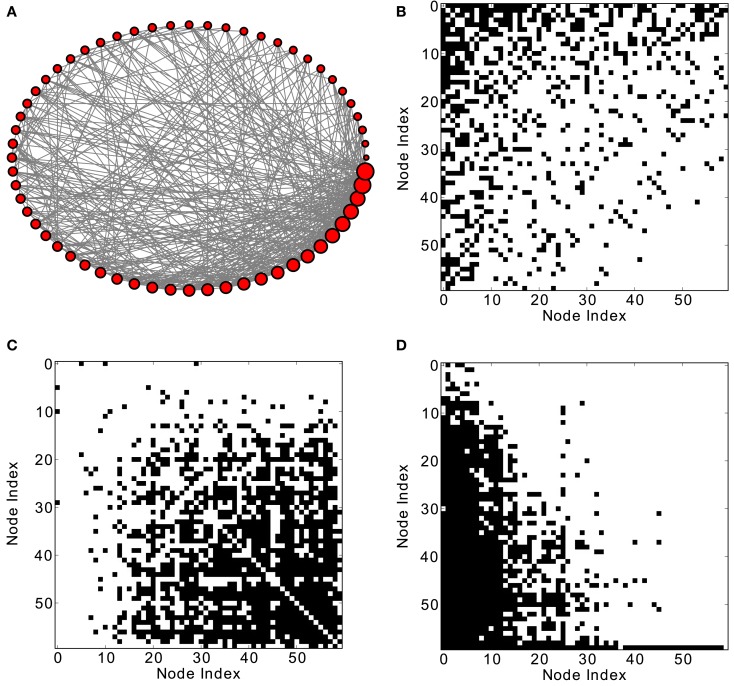
**Excitation patterns of a Barabási–Albert (BA) scale-free graph with 60 nodes and 800 links. (A)** Graphical representation of the network (the node degree is represented by the size of the nodes) and **(B)** adjacency matrix. Analysis of excitations: **(C)** average co-activation matrix with a threshold of 0.46 and **(D)** average co-activation matrix at time delay ±1 and threshold 0.3. All matrices are sorted according to the node degree of the adjacency matrix, from high to low degree.

Given the setup of the discrete dynamic model and a refractory period of one time step, the commonly observed period of node activation is three (for a detailed discussion of the mechanisms leading to the periodicity of node activations see Section 3.3). At this typical frequency, one can consider network activation patterns that arise from the co-activation of the nodes at zero delay, as well at a delay of one (see Figures 1C and **D**). Given the period length of three, co-activations at a delay of one time step are equivalent to co-activations at a delay of minus two time steps. Therefore, it is sufficient to display the zero-delay co-activations as well as co-activations for a delay of plus or minus one time step. Such diagrams, also including the adjacency matrix as well as visualizations of the respective networks, are given in Figure 1 and in subsequent figures.

Instantaneous co-activations label nodes which are jointly activated. This activation may arise from common input to the nodes as well as from independent inputs. The specific shape of the joint node excitation pattern may allow inferences on the likely input of nodes. By contrast, co-activations at plus or minus one time step delay reveal the apparent flow of excitations in the network. This apparent pattern does not necessarily represent causal signal flow, because the activation of a node may be caused by any of several active neighbors that are linked to the node. This additional contribution would be smaller in sparser networks. Moreover, an apparent transfer of excitations may also occur in nodes that are not themselves linked, but are targeted by a common input that affects them at a delay difference of one time step. Thus, the networks representing causal flow of excitations are a subset of the co-activation patterns at one time step delay. In a bidirectional network, one might expect that the average transfer of excitations from any node *i* to *j* is the same as from *j* to *i*; however, we found that there are network topologies that lead to an asymmetrical excitation flow between nodes.

**Figure 2:**
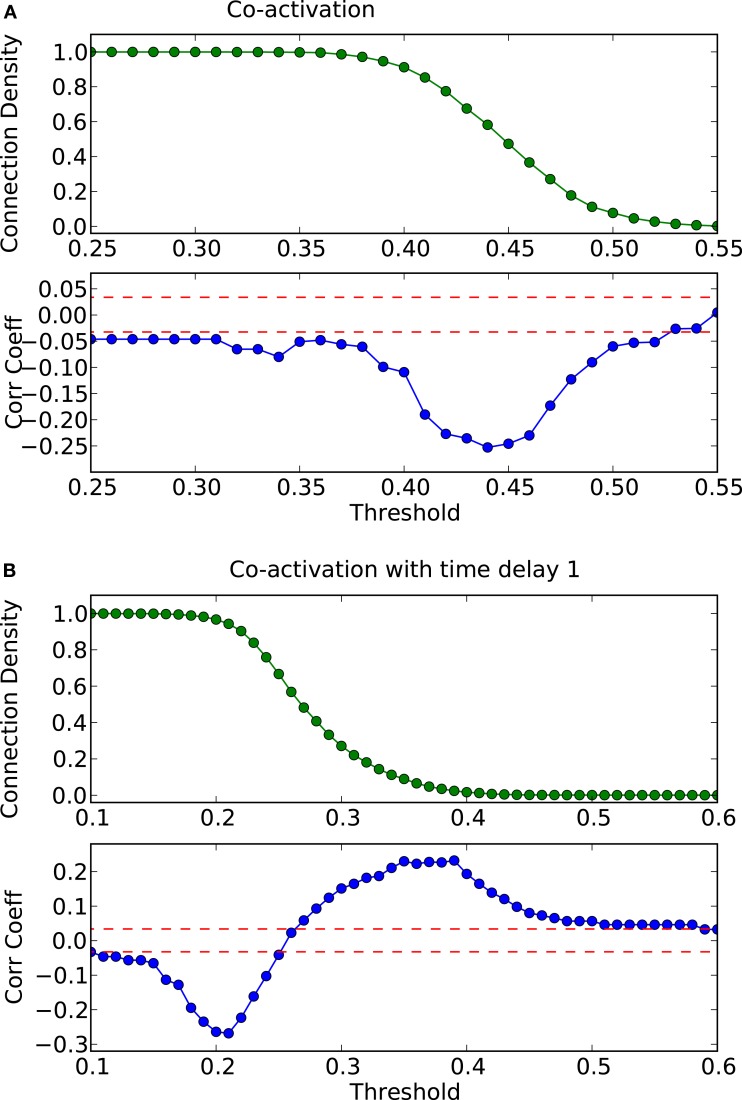
**Threshold dependence of the correlation coefficient between the adjacency matrix and the co-activation matrix** [**(A)**-lower panel, in blue] for a BA graph. The same for the co-activation matrix with time delay ±1 [**(B)**-lower panel, in blue]. The value of the average correlation coefficient (±the standard deviation) of binary random sequences is plotted in red. Connection density of the co-activation matrix [**(A)**-top panel, in green] as a function of the threshold, and the same graph for the co-activation with time delay ±1 [**(B)**-top panel, in green].

**Figure 3:**
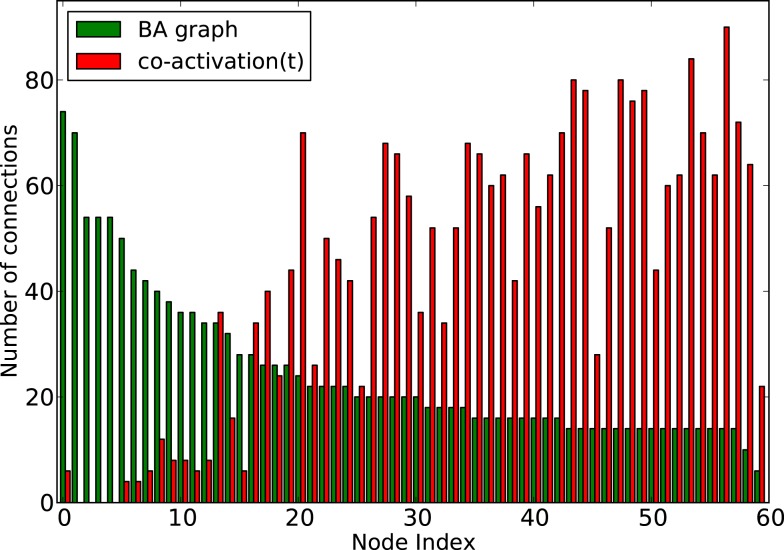
**Degree of the nodes (sorted by nodes’ degree in the adjacency matrix, from high to low) as a function of the node index, for a BA graph (in green) and its co-activation matrix at threshold 0.46 (in red)**.

How do global features of the structural network topology affect the co-activation patterns at different delays? To analyze the patterns, we converted the average co-activation matrix into a functional “adjacency” matrix by thresholding it into zeros and ones and contrasted it with the structural adjacency matrix. We calculated the Pearson correlation coefficient between the adjacency and the thresholded co-activation matrix to quantify the relation (as described in Section 2.3). In Figure 2A, we plotted the evolution of the correlation coefficient as a function of the threshold for a scale-free network. Apparent is a clear anti-correlation for intermediate values of the threshold, when the connection density of the co-activation matrix is approximately 0.5. We included in this figure the average correlation coefficient of binary random sequences with the same dimension of the adjacency matrix. Figure 3 shows a different representation of the anti-correlation. Here, the nodes’ degrees of the adjacency matrix are compared with the nodes’ degrees of the thresholded co-activation matrix. This plot clearly shows the anti-correlation of structural and functional connectivity for these dynamics, demonstrating that nodes which are connected in the structural graph are less co-active, and vice versa.

In scale-free networks, hubs are the highly connected central nodes of the network that re-distribute activity, forming the starting point of concentric, network-wide waves (Müller-Linow et al., 2008). Thus, most network nodes receiving direct, common input from the hubs are co-activated at the same time (Figure 1C). Therefore, the co-activations are anti-correlated to the structural adjacency matrix (Figure 2A). This anti-correlation results from the fact that, due to the discrete model, linked nodes which transfer excitation from one to another cannot be excited at the same time. Interestingly, there is a difference for excitations moving toward the hubs or away from them (shown as co-activations at positive or negative delays, upper-lower diagonal in Figure 1D). Thus, there is an asymmetry for the two spreading directions in hub networks, which is clearly apparent in the delayed co-activation matrix (Figure 1D), even when all network connections are bidirectional. Therefore, in this case we observed a positive correlation for connections directed away from the hub (Figure 2B).

**Figure 4:**
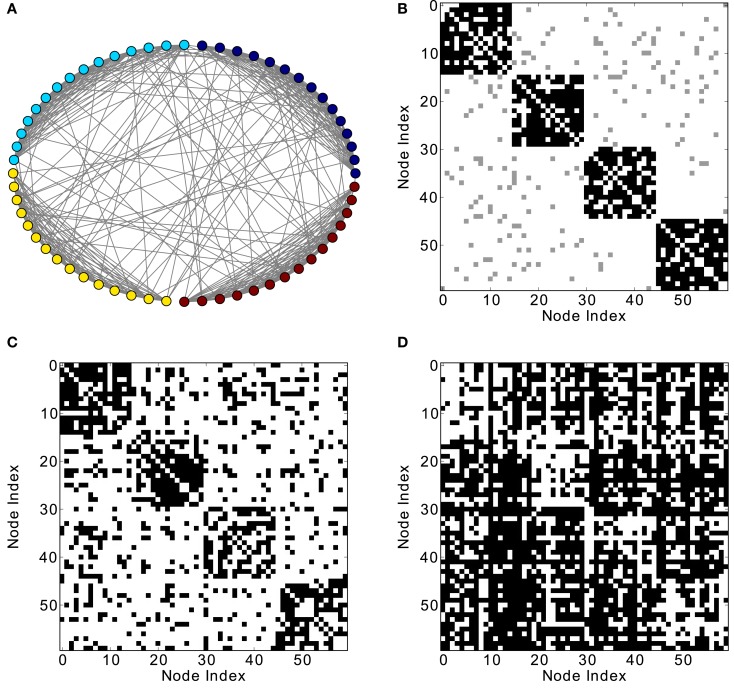
**Different network representations of a modular graph with 60 nodes and 800 links. (A)** Graphical representation of the network (the color of the nodes represents membership to different modules) and **(B)** adjacency matrix (intra-module links are represented in black and inter-module links are represented in gray). Analysis of excitations: **(C)** average co-activation matrix with a threshold of 0.44 and **(D)** average co-activation matrix with time delay ±1 and threshold 0.28.

**Figure 5:**
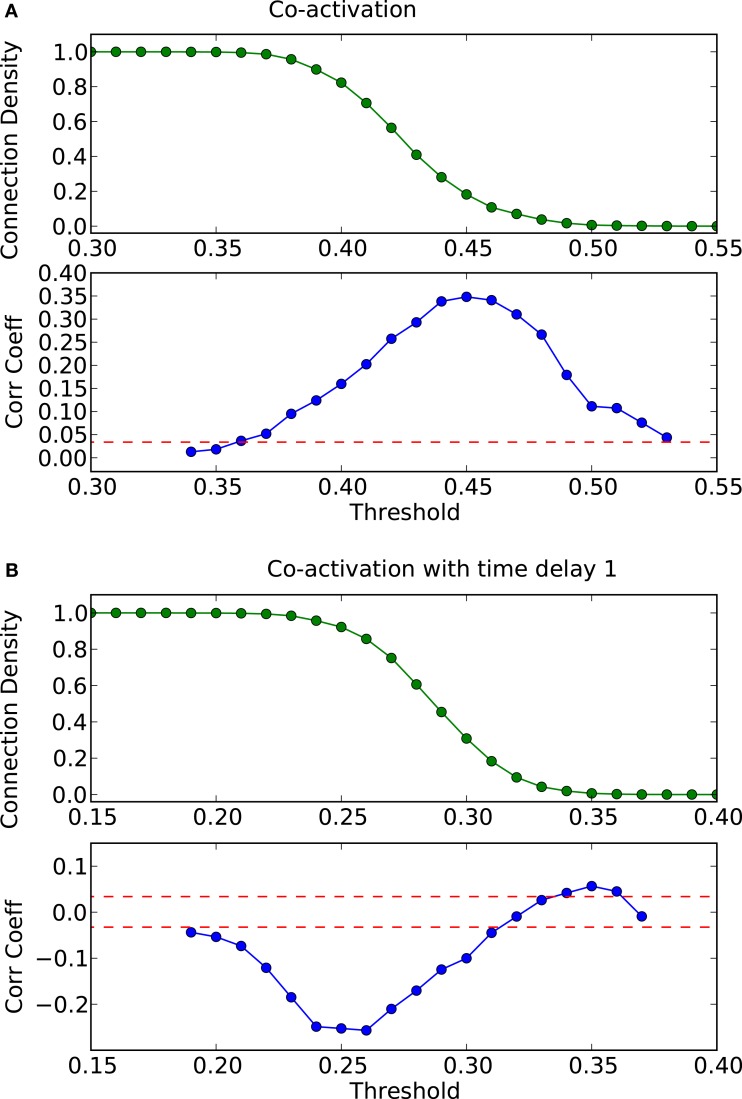
**Threshold dependence of the correlation between the adjacency matrix and the co-activation matrix** [**(A)**-lower panel, in blue] for a modular graph; same for the co-activation matrix with time delay ±1 [**(B)**-lower panel, in blue]. The value of the average correlation coefficient (±the standard deviation) of binary random sequences is plotted in red. Connection density of the co-activation matrix [**(A)**-top panel, in green] as a function of the threshold, and the similar graph for the co-activation with time delay ±1 [**(B)**-top panel, in green].

In modular networks, the modular organization is reflected in the co-activation patterns (Figure 4C), which now have a positive correlation with the adjacency matrix of the underlying network (Figure 5A). In other words, a modular network organization can override the anti-correlation between the co-activations and the structural adjacency matrix. Patterns at delay one (Figure 4D), however, are not modular, as they reflect longer-range network interactions that go beyond the interactions within modules (see Figure 5B).

**Figure A1:**
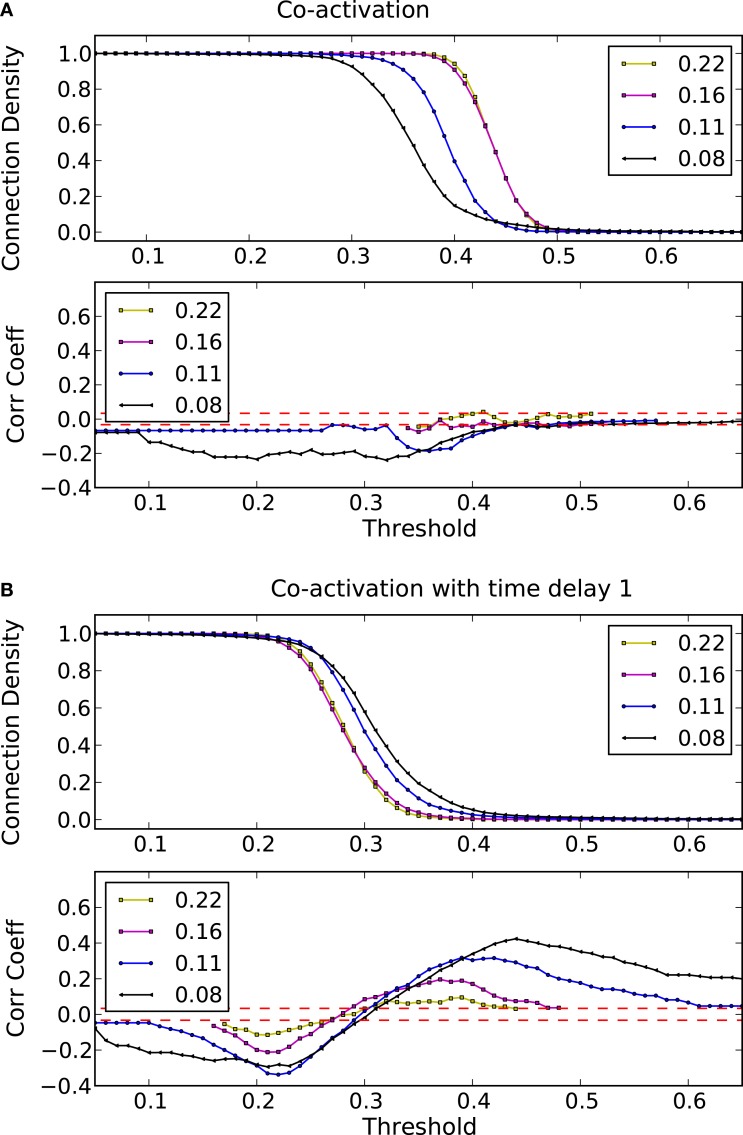
**Threshold dependence of the correlation coefficient between the adjacency matrix and the co-activation matrix** [**(A)**-lower panel] for a Erdős–Rényi (ER) graphs with different connection density; same graph for the co-activation matrix with time delay ±1 [**(B)**-lower panel]; in red is plotted the value of the average correlation coefficient (± the standard deviation) of binary random sequences. Connection density of the co-activation matrix [**(A)**-top panel] as a function of the threshold, and a similar graph for the co-activation matrix with time delay ±1 [**(B)**-top panel].

Due to the lack of a specific organization in ER-random graphs, the co-activations at different delays are also random. Nonetheless, these co-activations are anti-correlated to the adjacency matrix of the underlying network for sparser graphs (Figure A1A in the Appendix). For intermediate values of the connection density, the co-activation with delay is positively correlated with the adjacency matrix (Figure A1B in the Appendix). For these intermediate values, the co-activation highlights the links that are used to transfer excitations in the network.

**Figure 6:**
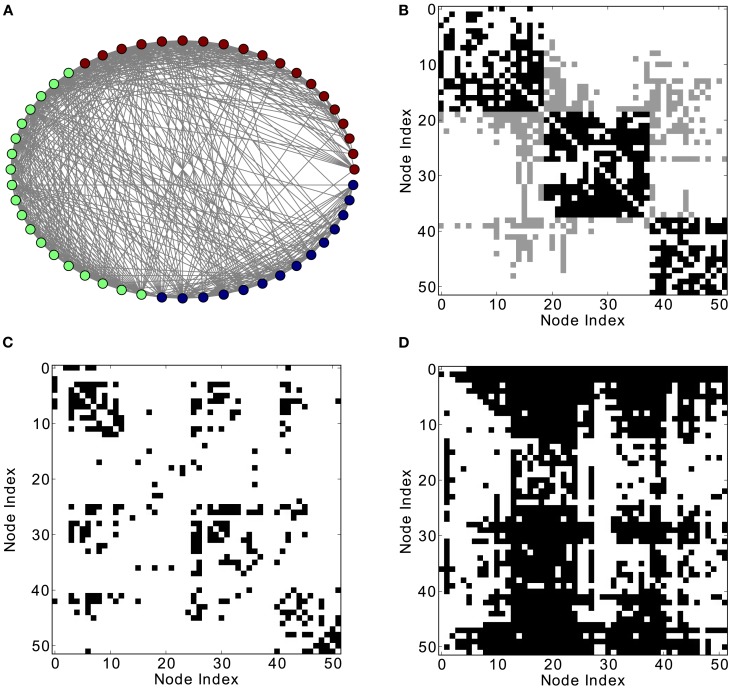
**Different network representations of the cortical connectivity of the cat (52 nodes and 820 links)**. **(A)** Graphical representation of the network (the color of the nodes represents membership to different modules) and **(B)** adjacency matrix (intra-module links are represented in black and inter-module links are represented in gray). Analysis of excitations: **(C)** average co-activation matrix with a threshold of 0.46 and **(D)** average thresholded co-activation with time delay ±1 and threshold 0.28.

**Figure 7:**
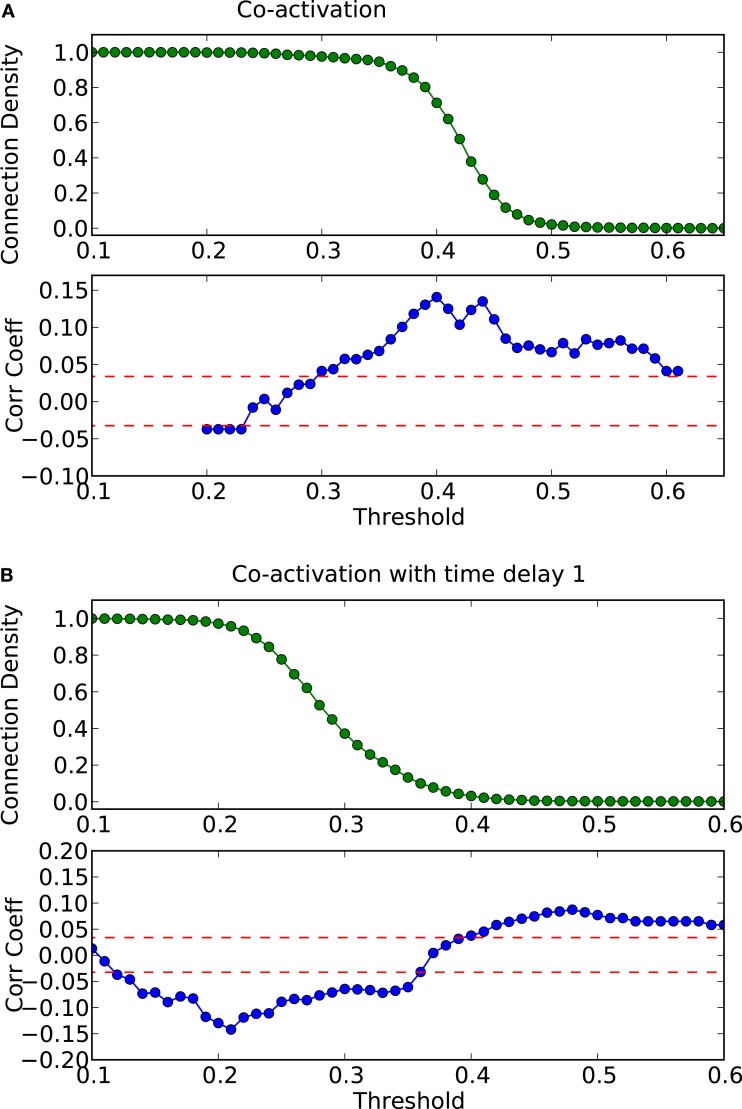
**Threshold dependence of the correlation between the adjacency matrix and the co-activation matrix** [**(A)**-lower panel, in blue] for the cat cortical network; same graph for the co-activation matrix with time delay 1 [**(B)**-lower panel, in blue]. The value of the average correlation coefficient (± the standard deviation) of binary random sequences is plotted in red. Connection density of the co-activation matrix [**(A)**-top panel, in green] as a function of the threshold, and the same graph for the co-activation matrix with time delay ±1 [**(B)**-top panel, in green].

**Figure 8:**
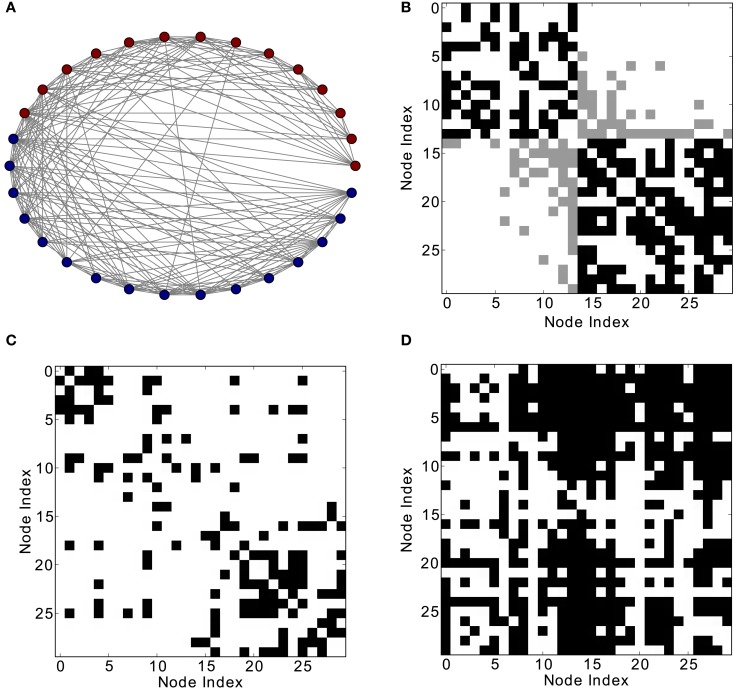
**Different network representations of the macaque visual cortex (30 nodes and 311 links)**. **(A)** Graphical representation of the network (the color of the nodes represents membership to different modules) and **(B)** adjacency matrix (intra-module links are represented in black and inter-module links are represented in gray). Analysis of excitations: **(C)** average co-activation matrix with a threshold of 0.45 and **(D)** average co-activation matrix with time delay ±1 and threshold 0.28.

**Figure 9:**
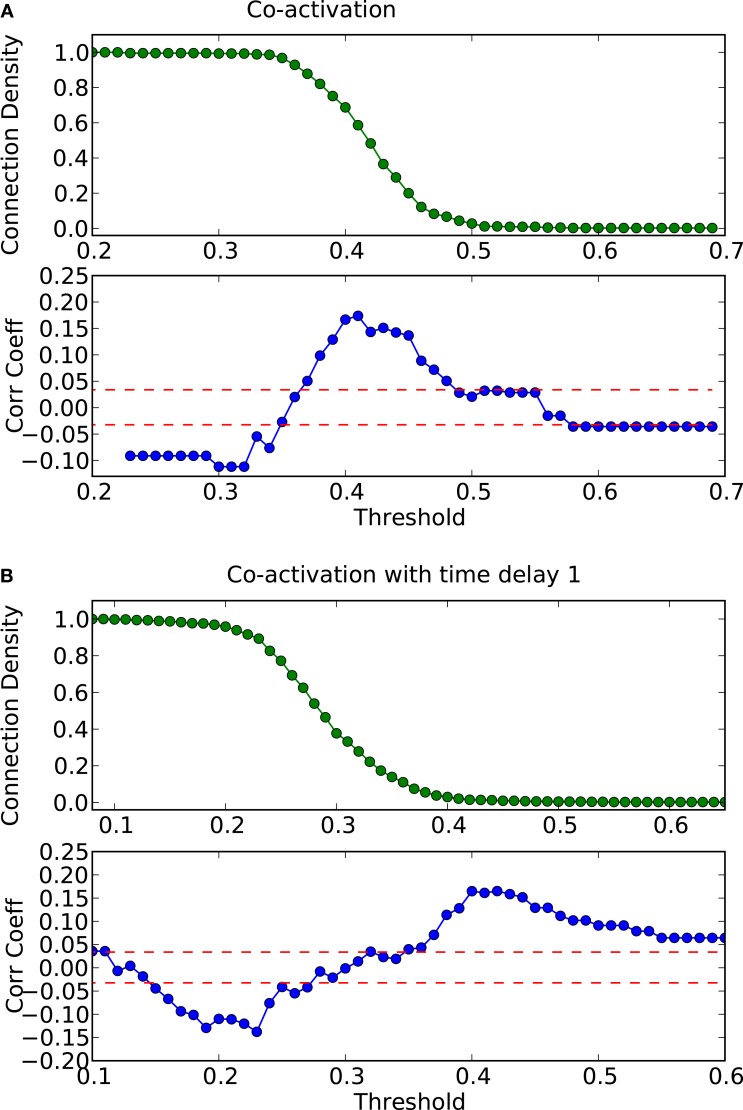
**Threshold dependence of the correlation between the adjacency matrix and the co-activation matrix** [**(A)**-lower panel, in blue] for the macaque visual cortex network; same graph for the co-activation matrix with time delay ±1 [**(B)**-lower panel, in blue]; the average correlation coefficient of binary random sequences is plotted in red (± the standard deviation). Connection density of the co-activation matrix [**(A)**-top panel, in green] as a function of the threshold, and the same graph for the co-activation with time delay ±1 [**(B)**-top panel, in green].

**Figure 10:**
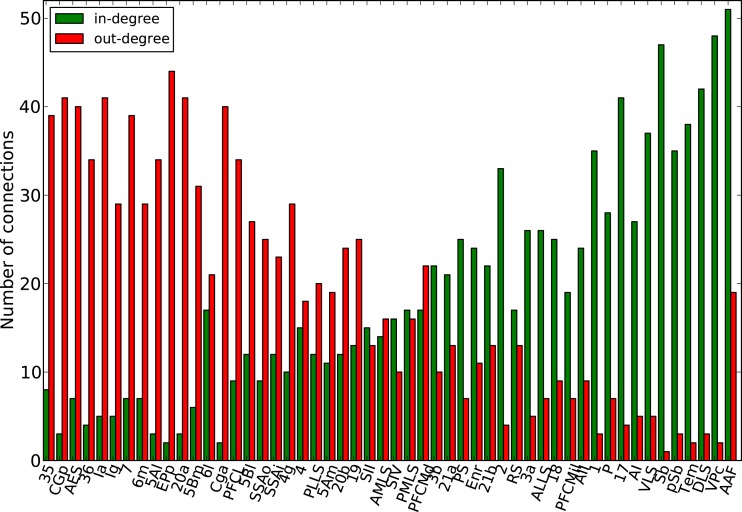
**Functional nodes’ degrees of the cat cortical network as a function of the node areas (sorted by nodes’ degree of the adjacency matrix, from high to low) for the delayed co-activation matrix with threshold 0.3**. In-degree is plotted in green, and out-degree in red.

**Figure 11:**
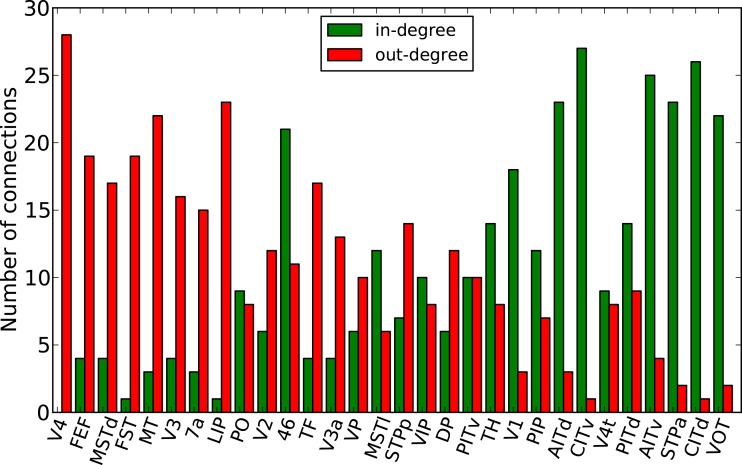
**Functional nodes’ degrees of the macaque visual cortex network as a function of the node areas (sorted by nodes’ degree of the adjacency matrix, from high to low) for the delayed co-activation matrix with threshold 0.3**. In-degree is plotted in green, and out-degree in red.

Finally, the biological examples of cortical networks of the cat and monkey brain show patterns that appear to arise from combining modular and hub features. On the one hand, the co-activations delineate modules reminiscent of those of the underlying structural networks. Therefore, the core activations are positively correlated with the structural adjacency matrix for both networks (Figures 7A and 9A). On the other hand, co-activations at a delay of plus or minus one time step are strongly asymmetrical (Figures 6 and 8) and positively correlated with the adjacency matrix (Figures 7B and 9B), similar to the delay co-activations for the hub network (Figure 1), and unlike the delay co-activations of the random-modular network (Figure 4). In particular, hub-like network nodes possess a high out-degree for the delay co-activations, marking them as the starting point of wave-like spreading of excitation, while nodes with few connections possess a high in-degree for delay co-activations, indicating them as recipients of excitation waves (Figures 10 and 11). Thus, area nodes in the biological networks are assigned dynamic roles, such as organizer of modular co-activation or sender versus receiver of excitation, that reflect aspects of their topology, such as modular membership and degree.

**Figure 12:**
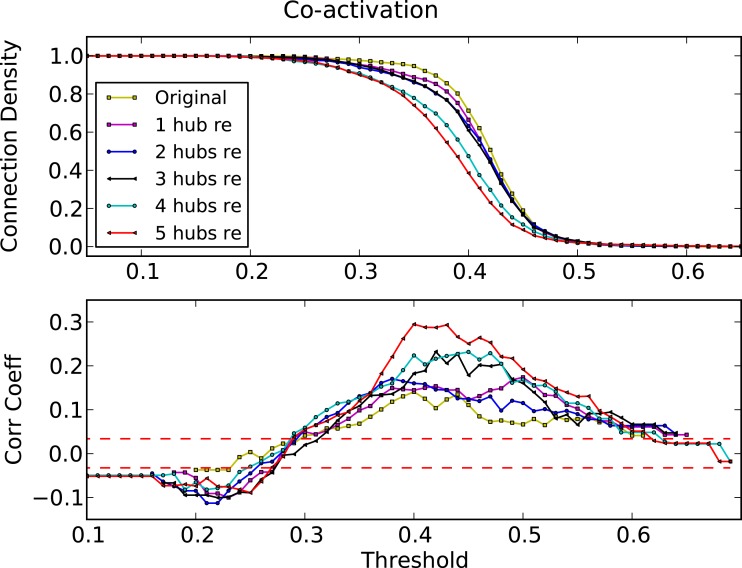
**Threshold dependence of the correlation between the adjacency matrix and the co-activation matrix (lower panel) for the cat cortical network after eliminating one to five hub nodes**. Connection density of the co-activation matrix (top panel) as a function of the threshold.

To verify the apparent dynamic competition between hub features and the modular organization in the biological networks, we eliminated hub nodes in the cat cortical network (Kaiser et al., 2007b; Sporns et al., 2007) and re-simulated the dynamics. We consecutively eliminated five nodes (and all their connections), starting with the highest degree node. Figure 12 shows the corresponding correlation coefficients between the co-activation matrix and the adjacency matrix. Upon the removal of hub nodes, the correlation coefficient increased to similar values as observed in the artificial ER-modular graph (Figure 5).

### 3.2 Link between macroscopic and microscopic perspective

So far, we focused on the macroscopic perspective, analyzing co-activation matrices obtained from numerical simulations of our deterministic minimal model of excitable dynamics. We observed a systematic impact of some global topological features on these co-activation matrices.

In this section, we “zoom into” these dynamics and explore the more microscopic foundations of sustained activity. As mentioned in Section 1, similar explorations with different dynamical models are found in Qian et al. (2010b) and McGraw and Menzinger (2011). Our deterministic model allows us to obtain a deeper microscopic understanding of sustained activity that serves as a (deterministic) basis for explaining similar effects in time-continuous and stochastic simulations, as well as experimental data.

For dense graphs, the generally observed average excitation density is 1/3, due to the collective period-3 oscillation. Here we explore sparser graphs to better understand the topological prerequisites of sustained activity. In many ways, the deterministic model is a very schematic toy model version of an excitable cell or population.

**Figure 13:**
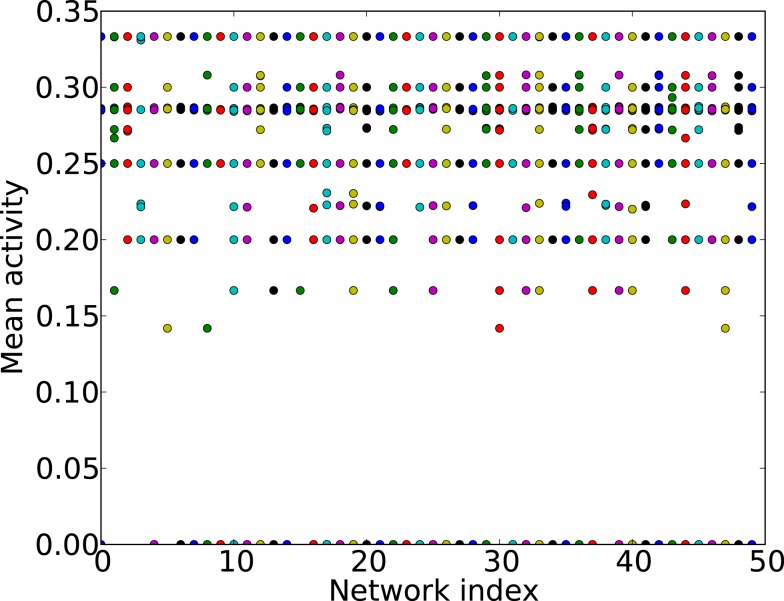
**Mean activity (*y*-axis) for different initial conditions and different ER graphs (*x*-axis, network index)**. All the networks have 60 nodes and 250 links.

For sparse graphs, the average excitation density depends on the initial conditions, see Figure 13. If the inter-excitation interval (or period) for all nodes is three times steps, the average excitation density is 1/3, for four times steps the mean activity is 1/4, and for five time steps 1/5 (Figure 13). It is also possible that some nodes present a period of three times steps followed by a period of four times steps; in this case the mean activity will differ from the values mentioned before. We use the mean activity measure as a proxy for periodicity of the global activity.

**Figure 14:**
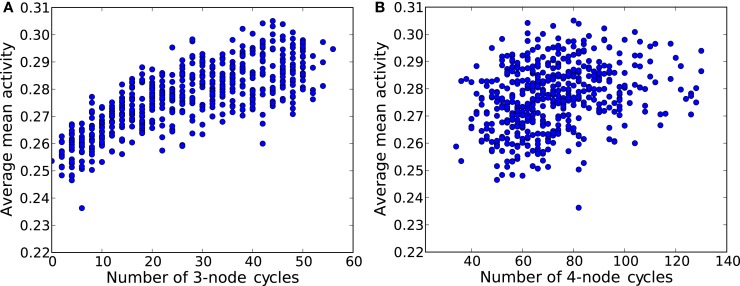
**Average mean activity for different ER graphs versus (A)** the number of 3-node cycles and **(B)** the number of 4-node cycles. Each network is a ER graph with 60 nodes and 250 links.

The first step toward this microscopic interpretation is given by Figure 14, where the average mean activity (average over many initial conditions) as a function of the number of elementary 3-node (Figure 14A) and 4-node cycles (Figure 14B) is shown. We observe a clear positive correlation of the mean activity and the number of 3-node cycles, while the number of 4-node cycles has no impact on this measure.

Even on this purely statistical level, small topological devices such as 3-node and 4-node cycles have an impact on the amount of sustained activity observed in the network. Furthermore, dense networks seem to favor smaller devices.

In the following sections we study these individual devices under our dynamics in more detail. In particular, we constructed basins of attraction for the different possible asymptotic states and discussed the effect of embedding these devices into larger networks.

### 3.3 Small devices

The key observation from the previous section is that indeed a systematic relationship between the number of 3-node cycles and the mean activity can be identified. Therefore, we now analyze the smallest network components that are capable of producing sustained activity on their own. Specifically, we explore “small topological devices,” such as 3-node cycles (“triangles”) and 4-node-cycles (“squares”).

We here extend the work from Carvunis et al. (2006), in which the role of triangles where the three vertices are, respectively, in the state *E*, *S*, and *R* has been evidenced: they behave as dynamic motifs of period 3 totally insensitive to the surrounding activity, that is, as robust pacemakers.

**Figure 15:**
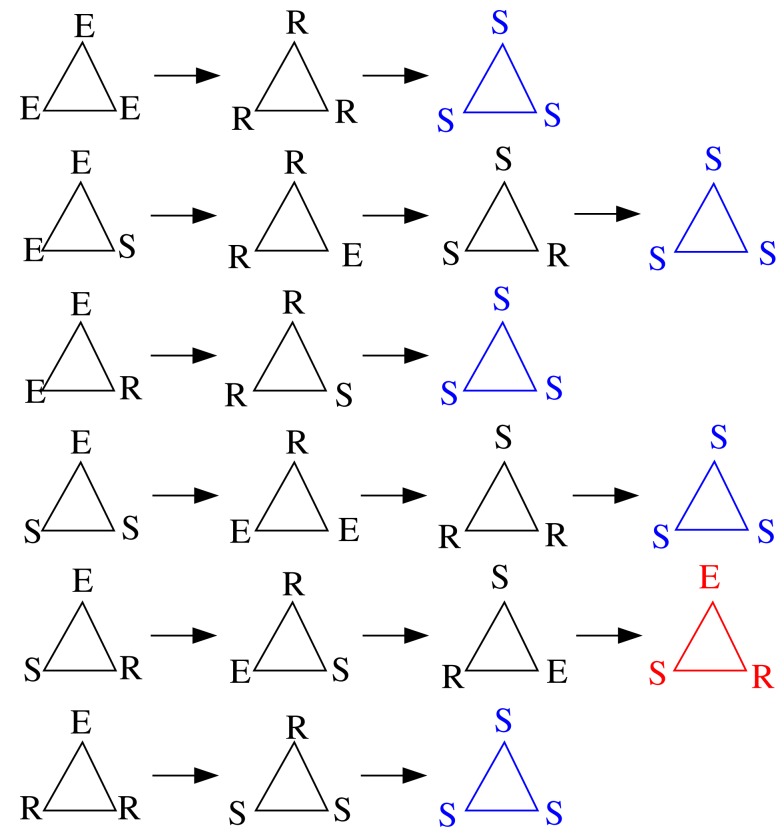
**Time course (from left to right) of triangles for all possible initial conditions with at least one excitation (top to bottom; omitting symmetrical ones) with the model described in Section 2.1**. The last time step shown in each row allows to discern the asymptotic behavior: all-susceptible steady state (blue) and sustained periodic activity (red).

For such small devices, like triangles or 3-node cycles, we can now indeed enumerate all possible initial conditions. Figure 15 displays these possibilities (not showing symmetrical conditions) and follows them through time. Of the nine cases, only two initial configurations settle into sustained periodic activity. The only two configurations that produce sustained activity are the *ESR* and *ERS* in the triangle.

#### 3.3.1 Preliminary considerations: necessary conditions for sustained activity

In this model the following thought experiment is possible that will serve as a guideline for the remaining parts of our investigation: We can formally enumerate all possible initial conditions and observe how many will settle into a periodic asymptotic behavior. A question central to our investigation is, thus, which topological properties lead to an increase in the number of sustained outcomes?

The general framework of spatiotemporal pattern formation, and in particular spiral waves, which can be seen as the spatiotemporal equivalent of sustained activity, can provide some interesting insights into contributors to sustained activity in general and, more specifically, the mapping of initial conditions to asymptotic behavior. Qualitatively speaking, the cases where an odd number of “susceptible” – “excited” – “refractory” states neighbors appear in the initial conditions can serve as a mean-field-like expectation of the number of successful initial conditions, accounting for a certain portion of the connectivity dependence (due to the total number of neighbors).

When edges are topologically undirected, both forward and backward propagation are possible when introducing an excitation from outside in a chain of susceptible nodes. Having a triplet of neighbors with the initial settings *S*–*E*–*R* induces directionality in the excitation propagation. The presence of a refractory stage prevents backward propagation, and makes the paths dynamically directed. Coexistence of two excitations cycling along the closed paths are only possible if the cycle is properly initialized (and it is not robust with respect to the surrounding activity). Otherwise, the incoming excitations (from outside) propagate in both directions and annihilate those propagating behind; only the most advanced survive, which lead to an accelerating phase slip when occurring. Once in a state of cycling excitation, a single external input can only shorten the period. This leads to an important caveat: cycle length does not always corresponds to inter-spike interval (accelerating phase slips are possible upon the involvement of external excitations).

The idea is that an excitation neighbored by a refractory and a susceptible element is a seed configuration of a propagating “wave” in the graph. Let us consider a ring of some not-too-small length *l*. A single excitation placed in an otherwise susceptible graph will generate two such “wave fronts” that will meet again after ⌊*l*/2⌋ time steps and this transient activity will die out with the graph settling into an all-susceptible state due to the “annihilation” of the two “wave fronts.”

Let us now consider the case of a single excitation placed into this ring graph with a single refractory neighbor, while the remaining *l* − 2 nodes are all in their susceptible state. In this case, the “wave front” will propagate unidirectionally along the ring and if the length *l* is larger than the (deterministic) refractory period *r*, we will have sustained activity. This is in strong analogy to the core region of a spiral wave in spatiotemporal pattern formation.

Next, we consider the case where in addition to the previous initial conditions one of the remaining *l* − 2 − *r* nodes (with the exception of the other direct neighbor of the refractory state, as well as the *r* − 1 next-to-nearest neighbors in that direction) is initially in the excited state. In this case we have a unidirectionally propagating wave front, like before, and additionally in some distance two wave fronts emanating from the other excited element and propagating in opposite directions. The first will annihilate with one of the others, while the remaining wave front will persist. Thus, a very large number of deviations from the successful initial condition described above (based on the seed of “susceptible”, “excited” and “refractory” states neighbors) will also be successful and lead to asymptotic sustained activity.

**Figure 16:**
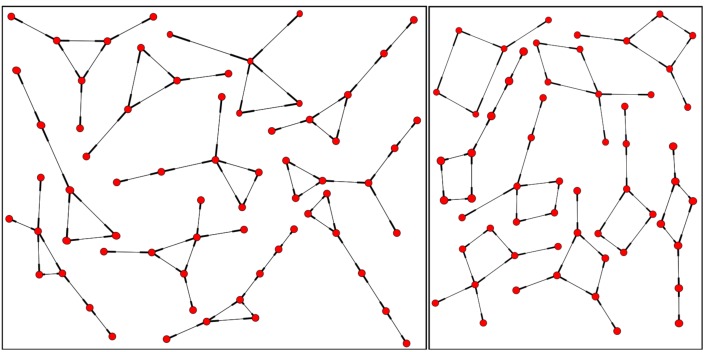
**Some triangle (left) and square (right) embeddings, local neighborhoods within the network with degree 2, 3 up to degree 4**.

As a next step, we verify for the triangle that the background does not matter as shown in Carvunis et al. (2006). It is clear that once the triangle settled into periodic activity, it cannot be disrupted by random excitations. A possible source of such random excitations (apart from spontaneous activity) can come from the embedding of such a device in a larger network. Having observed that this sustained activity in a triangle, once established, will persist, even when the triangle is located in a network, the next natural question is whether the basin of attraction for the periodic asymptotic behavior changes due to the embedding of the device in the network. We considered various such embeddings, with five, six, and seven nodes (Figure 16). We computed the total number of initial conditions that lead to sustained activity from all possible initial configurations when we consider one to four excitations in the devices. The success of an initial configuration does not depend on the number of initial excitations nor on the triangle embedding, but only whether the triangle is initially in the *ESR* configuration or not. We confirmed our numerical observation by comparing the results with the analytical formula described in the Appendix.

**Figure 17:**
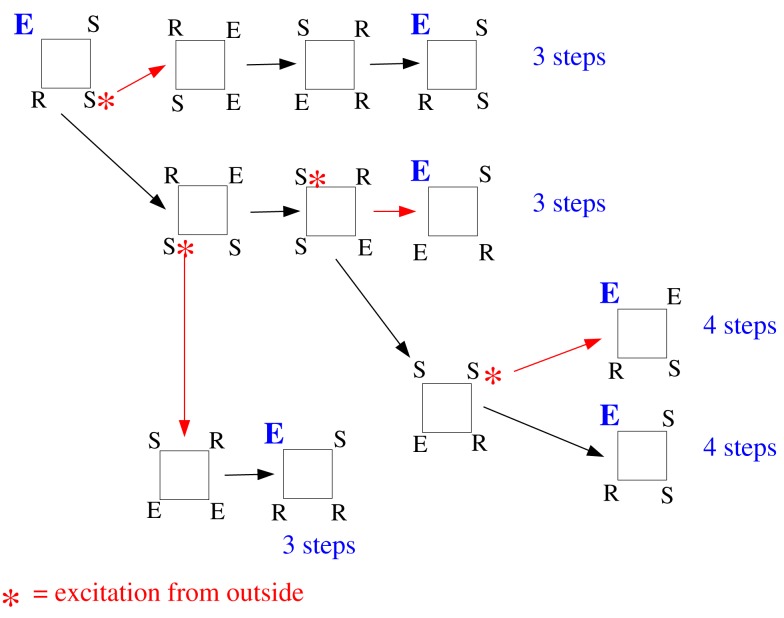
**Effect of spontaneous excitations perturbing the time course of a successful initial condition on a square (consisting of a single triplet of "susceptible", "excited" and "refractory" states neighbors and one susceptible node**. “Noise” excitations are marked by an asterisk. The first noise excitations has already been added to the initial configuration (left-hand side; turning one of the two susceptible elements into an excitation). Toward the right, each row explores one possibility of time course, partly depending on additional noise events. The numbers indicated at the end of each row are the time steps until the initial configuration is reached again.

We repeated the same general discussion for a square. Again, we counted the successful initial conditions and, like in the case of the triangle, we analyzed how robust this sustained activity is with respect to spontaneous excitations or outside excitations (represented by asterisks in Figure 17) when the square is embedded in a larger network.

The configuration *ESSR* of the square also produces sustained activity, whatever the background (which only affects the recurrence time and which configuration is actually recurrent, but not the fact that there is recurrence, i.e., sustained activity). In Figure 17 we have taken into account all the instances, whether the background brings an additional excitation (the red-star excitation from outside arriving at other S locations produce the same effect as the propagation of excitation within the motif) or not. As we mentioned before, we consider the inter-excitation interval for one node as the period of node excitation.

The first observation is that, as in the case of the triangle, sustained activity on a square device is also robust against such perturbations. The second observation is of importance for looking deeper into the structure of the output signal in time: While the time output of the triangle is strictly periodic, the square can display phase slips, where the regular period-4 output is sometimes disrupted (due to the noise-like excitation events) by other inter-excitation intervals in the time courses of the nodes.

The square is the smallest device capable of displaying the general phenomenon of such phase slips already hinted on the thought experiment discussed previously. We can now imagine the square being embedded in the network in a way that such excess excitations arrive systematically at the right moment. In this case, the embedded device would yield, e.g., a period-3 oscillation in spite of the underlying cycle length of four. This is a first example where the periodicities observed in the dynamics do not directly match the periodicities dictated by the “hardware,” i.e., the cycles present in the graph.

**Figure 18:**
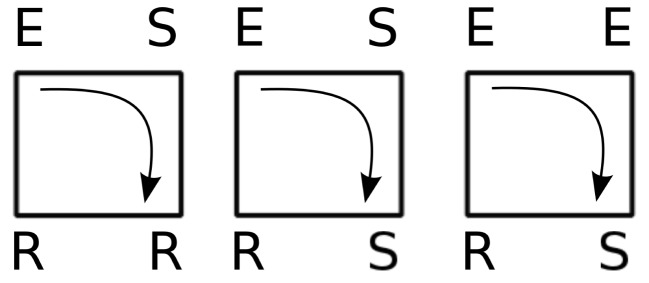
**Initial conditions that lead to sustained activity for an isolated square**.

As we did before for the triangles, we analyzed how the basin of attraction of the squares changes when we consider some additional nodes coupled to it (Figure 16). In analogy to the combinatorial prediction of the successful initial conditions for the triangle, we can also predict the numerical results (see the Appendix for details). As before for the triangle, the embedding does not affect the basin of attraction of the sustained activity beyond combinatorial effects. All the initial configurations that lead to sustained activity for an embedded square (Figure 18), as we mentioned in the thought experiment, contain an excited node neighbored by a refractory and a susceptible element.

## 4 Discussion

In our study, we started addressing the question of what topological aspects of an excitable network are responsible for characteristic features of the network dynamics. Starting from an established three-state model of neuronal activity that forms a stochastic cellular automaton (see Müller-Linow et al., 2006, 2008; Hütt and Lesne, 2009; Hütt et al., 2012), the model was taken to the limit of few spontaneous excitations and high recovery probability [i.e., f → 0 and p → 1 in the notation of the model from Müller-Linow et al. (2006)], resulting in a deterministic discrete excitable node model that is simple enough for an exhaustive analysis of small networks. With the help of this model, we explored the topological determinants of periodic, self-sustained activity as well as the large-scale co-activation patterns that can be observed in random, modular and hub networks as well as in networks of cortico-cortical connections of the mammalian brain.

The present deterministic model is a cellular automaton (CA) and can therefore also be discussed in the context of CA on graphs. Cellular automata (CA) have been used in a vast number of investigations to explore the emergence of complex patterns from simple dynamic rules. Originally defined on regular lattices (Wolfram, 1983), they have also been studied on more complex topologies (Traub et al., 1999; Amaral et al., 2004;Marr and Hütt, 2005, 2009) and in noisy environments (Moreira et al., 2004; Marr and Hütt, 2006). The principal goal of discussing CA on graphs is to explore the relationship between network architecture and dynamics from the perspective of pattern formation. Also for CA on graphs, the Wolfram classes (Wolfram, 1983, 1984) are a helpful and established means of characterizing observed dynamic behaviors (see, e.g., Marr and Hütt, 2005). It seems that the sequential nature of states in our cellular automaton (each cell cycles through the states in the order *S* → *E* → *R*) and the “diffusive” neighborhood coupling restrict the Wolfram classes to *I* (fixed point) and *II* (periodic). We do not observe Wolfram classes *III* (chaotic) and *IV* (complex). However, we have no formal argument ruling out these dynamics. Extending the formalism from Marr and Hütt (2009) to three states, enumerating all possible CA of that type (with the CA discussed in the present paper being represented as one example) and then studying how the number of complex (Wolfram class *IV*) dynamics changes with network architecture would be a very informative investigation.

We found that features of network dynamics are based on topological network attributes at different scales. Such scales are represented by isolated small topological “devices” (cycles of 3 and 4 nodes) on the microscopic scale, the same devices embedded in a particular “network environment” at the mesoscopic scale, and large-scale network features, such as modules or hubs, at the macroscopic scale. While devices comprising only a few nodes (e.g., cycles of 3 and 4 nodes) are the origin of sustained network activity and the periodicity of node activation patterns, larger-scale topological features appear responsible for the average co-activation patterns of the networks.

Systematic relations emerged between functional and structural connectivity in different network topologies. As a general feature, simultaneous activation of linked nodes was suppressed in the model, due to the inclusion of a refractory phase. Therefore, nodes that are linked tend to be anti-correlated in their activity, in the sense that a node which transfers its excitation to a directly connected node turns refractory in the next step and, thus, is not active at the same time as its target. The anti-correlation of the co-activation matrix and the graph’s adjacency matrix was in particular observed for sparse random and hub networks.

However, it also emerged that this anti-correlation depends strongly on global properties of the network architecture. The minimal model allowed us to study systematically which topological properties of the network enhance or reduce this attribute. In particular, the suppression of joint activation of linked nodes can be “overwritten” in graphs with high clustering, such as encountered within the modules of a modular graph, due to the largely shared input of intra-modular nodes. In modular graphs, nodes are most frequently linked within the modules, forming cliques. Therefore, two connected nodes are also likely to share the same neighbors and, thus, frequently receive common input. As a consequence, the anti-correlation was strongest in sparse random networks, but was not observed in random-modular networks or the studied biological cortical networks, which are also modular (Hilgetag et al., 2000).

Several potential functions have been suggested for the modularity of brain networks. These functions include increased robustness against network damage such as through link lesions (Kaiser and Hilgetag, 2004), increased synchronizability, higher levels of limited sustained activity (Kaiser et al., 2007a; Kaiser and Hilgetag, 2010), and self-organized criticality (Wang et al., 2011) as well as increased dynamic complexity of neural systems (Sporns et al., 2000), based on the idea of higher integration of nodes within modules combined with their segregation between modules. The present findings provide an additional explanation for the modular organization of brain dynamics in neural systems (Zhou et al., 2006; Honey et al., 2007) and suggest that modular co-activation patterns are based on the common input of nodes within the structural modules of cortical networks.

Clearly, the generic anti-correlation between co-activation and connectivity is enhanced by the minimal nature of our model, in particular, the discrete time and the synchronous, deterministic updating. However, a contribution to co-activation that displays significant anti-correlation to the adjacency matrix might also be expected to be seen in more refined dynamic models. In the light of the fact that correlation networks yielding functional connectivity are frequently employed as data analysis and data representation strategies (Greicius et al., 2009; van den Heuvel et al., 2009), it is important to emphasize that, indeed, structural connectivity and functional connectivity can be anti-correlated, with the (structural) network architecture regulating the strength of the anti-correlation, for example, via clustering and modularity.

Further information on the propagation of excitations in the networks was derived from the delay co-activation matrix at plus or minus one time step. The strong asymmetry between the upper and lower triangles of this matrix for scale-free graphs is an indicator of the waves described in Müller-Linow et al. (2008): excitations propagate toward the hub(s) individually, while they emerge from the hub in a coherent fashion. Therefore, surprisingly, the large-scale topologic feature of a hub imparts a directionality on the structurally bidirectional links of an excitable network. This phenomenon is also apparent in a more graded fashion for the biological networks, where nodes with a high degree were the main sources of excitations, while low-degree nodes formed sinks. Thus, the biological networks combine dynamic features based on modules as well as hub-like nodes. While these cortical networks may lack very pronounced hubs in the sense of a scale-free network (Barabási and Albert, 1999), it is clear that they have a wide, non-random degree distribution (Kaiser et al., 2007b), and may possess hub-like nodes that were identified by topological analyses (Sporns et al., 2007; Zamora-López et al., 2010), lesion simulations (Kaiser et al., 2007b), or by dynamic exploration (Müller-Linow et al., 2008). The present study suggests another potential role of these highly connected, central network nodes, that they may serve as the origins of recurrent excitation waves in the cerebral network. Interestingly, these sources are not formed by primary cortical areas, but rather by areas removed from the sensory and motor periphery, underlining the importance of endogenous cortical sources for self-sustained dynamics, in contrast to dynamics evoked by external inputs. In particular in the cat (Figure 10), these excitation sources are formed by a group of multi-sensory, parahippocampal, and limbic areas. Perhaps it is no coincidence that such areas are also frequently suspected to be the origin of epileptic spreading in the human brain (Penfield and Jasper, 1954). In the monkey visual cortex (Figure 11), the sources of excitation waves are also formed by higher-order areas, particularly of the dorsal visual stream, that are some steps removed from visual input, while most areas of the ventral visual stream, particularly in the inferotemporal cortex, are recipients of excitation. It will be interesting to see if these patterns can also be identified in experimental resting-state data.

At the level of smaller topological devices, previous studies (Milo et al., 2004; Sporns and Kötter, 2004) showed that neural networks have a characteristic content of motifs, in particular of well-connected motifs. However, the exact functional roles of these motifs while embedded in networks have not been clear. Previous work using ODE modeling suggested that these topological features can play the role of dynamic pacemakers (Qian et al., 2010b). Here we confirmed this idea with the discrete model, which allowed us to investigate the dynamic behavior of these small circuits in an analytical and exhaustive way. We showed by numerical simulation that the frequency diversity decreases, and consequently, the mean activity increases, with the number of triangles in a graph. We also showed that an embedding of triangles and squares does not alter the basin of attraction of oscillatory behavior, that is, the number of initial conditions leading to self-sustained activity. Any initial condition containing *S*–*E*–*R* in the cycle (and none other) produces sustained oscillations. The only new phenomenon arising on this level are phase slips, such as period-3 contributions to the oscillations on squares, which we also observed.

In summary, the characteristic dynamics of prototypical graphs as well as biological neural networks appear to be shaped by a combination of topological features at different network scales. It will be a challenge for the future to link these local and global topological perspectives in understanding neural network dynamics and to confirm the present results in more intricate dynamical models, involving stochastic parameters as well as noise. Moreover, it has been claimed that pattern generation on neural graphs essentially relies on a well-chosen distribution of delays (Deco et al., 2009). It remains to be further explored if some of these patterns can also be reproduced in discrete models without delay.

## Conflict of Interest Statement

The authors declare that the research was conducted in the absence of any commercial or financial relationships that could be construed as a potential conflict of interest.

## Figures and Tables

**Figure A2:**
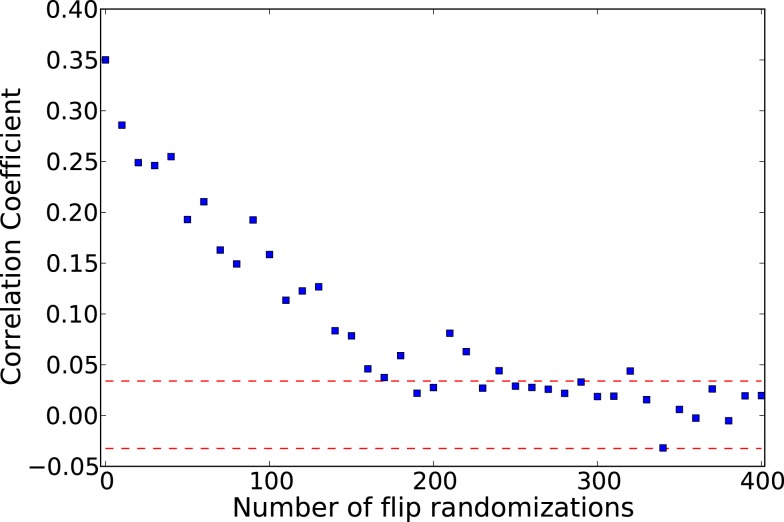
**Correlation coefficient between the adjacency matrix and the average co-activation matrix, for a fixed threshold, starting with the artificial ER-modular graph shown in Figure 4 and then randomizing the connections toward a ER-random graph**. The fixed threshold 0.45 was chosen where the correlation coefficient between the adjacency and the co-activation matrix of the ER-modular is maximal (see Figure 5A)

## Appendix

### Small devices

We investigated the dynamics of single small devices within their surrounding, and identified pacemakers, if any. That is, local motifs whose activity is able to control and sustain the activity of a larger subnetwork in which they are embedded. We started with 3-node and 4-node cycles. In Figure 16 we illustrate various such embeddings for 3-node and 4-node cycles.

The dependence of the percentage of successful initial conditions *s*(*n*,*k*) on the embedding (i.e., the number *k* of excess nodes beyond the device) and on the number *k* of excitations initially present can be easily understood by assuming that any successful initial condition requires a permutation of *SER* in the triangle. This leads to 3! configurations for the triangle. For a single excitation, we thus have 3! 2*^n^* successful initial conditions, where *n* is the number of excess nodes. With two excitations, a successful initial condition requires one of them to be in the triangle and the other in the excess nodes, yielding 3! *n* 2*^n^−1* cases. In general, *k* excitations lead to

(A1) $$3!\left(\begin{array}{c} n\\ k-1 \end{array}\right)2^{n-(k-1)}$$

successful cases. The normalization for each of the numbers is given by the total number of initial conditions with *n* + 3 nodes and *k* excitations, i.e.

(A2) $$\left(\begin{array}{c} n+3\\ k \end{array}\right)2^{n+3-k}$$

yielding

(A3) $$s(n,k)=\frac{3\left(\begin{array}{c} n\\ k-1 \end{array}\right)}{2\left(\begin{array}{c} n+3\\ k \end{array}\right)},$$

for the percentage of successful initial conditions. For the percentage of successful initial conditions *s*(*n*,*k*) in the case of an embedded square we obtain:

(A4) $$s(n,k)=\frac{2\left(\begin{array}{c} n+1\\ k-1 \end{array}\right)}{\left(\begin{array}{c} n+4\\ k \end{array}\right)}.$$

## References

[B1] Amaral L. A. N. Díaz-Guilera A. Moreira A. A. Goldberger A. L. Lipsitz L. A. (2004). Emergence of complex dynamics in a simple model of signaling networks. Proc. Natl. Acad. Sci. U.S.A. 101, 15551–15555 10.1073/pnas.0404843101 15505227 PMC524828

[B2] Barabási A. L. Albert R. (1999). Emergence of scaling in random networks. Science 286, 509–512 10.1126/science.286.5439.509 10521342

[B3] Binzegger T. Douglas R. J. Martin K. A. C. (2004). A quantitative map of the circuit of cat primary visual cortex. J. Neurosci. 24, 8441–8453 10.1523/JNEUROSCI.1400-04.2004 15456817 PMC6729898

[B4] Brunel N. (2000). Dynamics of networks of randomly connected excitatory and inhibitory spiking neurons. J. Physiol. Paris 94, 445–463 10.1016/S0928-4257(00)01084-6 11165912

[B5] Buchanan M. (2012). It’s a (stylized) fact! Nat. Phys. 8, 3–3 10.1038/nphys2255

[B6] Bullmore E. Sporns O. (2009). Complex brain networks: graph theoretical analysis of structural and functional systems. Nat. Rev. Neurosci. 10, 186–198 10.1038/nrn2618 19190637

[B7] Buzsáki G. (2006). Rhythms of the Brain. Oxford: Oxford University Press

[B8] Carvunis A. R. Latapy M. Lesne A. Magnien C. Pezard L. (2006). Dynamics of three-state excitable units on Poisson vs power-law random networks. Physica A 367, 595–612 10.1016/j.physa.2005.12.056

[B9] Deco G. Jirsa V. McIntosh A. R. (2011). Emerging concepts for the dynamical organization of resting state activity in the brain. Nat. Rev. Neurosci. 12, 43–56 10.1038/nrg2902 21170073

[B10] Deco G. Jirsa V. McIntosh A. R. Sporns O. Kötter R. (2009). Key role of coupling, delay, and noise in resting brain fluctuations. Proc. Natl. Acad. Sci. U.S.A. 106, 10302–10307 10.1073/pnas.0901831106 19497858 PMC2690605

[B11] Engel A. K. Fries P. Singer W. (2001). Dynamic predictions: oscillations and synchrony in top-down processing. Nat. Rev. Neurosci. 2, 704–716 10.1038/35094565 11584308

[B12] Erdős P. Rényi A. (1960). On the evolution of random graphs. Publ. Math. Inst. Hung. Acad. Sci. 5, 17–61

[B13] Felleman D. Van Essen D. C. (1991). Distributed hierarchical processing in the primate cerebral cortex. Cereb. Cortex 1, 1–47 10.1093/cercor/1.1.1 1822724

[B14] Fries P. (2005). A mechanism for cognitive dynamics: neuronal communication through neuronal coherence. Trends Cogn. Sci. (Regul. Ed.) 9, 474–480 10.1016/j.tics.2005.08.011 16150631

[B15] Galán R. F. (2008). On how network architecture determines the dominant patterns of spontaneous neural activity. PLoS ONE 3, e2148 10.1371/journal.pone.0002148 18478091 PMC2374893

[B16] Greicius M. D. Supekar K. Menon V. Dougherty R. F. (2009). Resting-state functional connectivity reflects structural connectivity in the default mode network. Cereb. Cortex 19, 72–78 10.1093/cercor/bhn059 18403396 PMC2605172

[B17] Hagberg A. A. Schult D. A. Swart P. J. (2008). “Exploring network structure, dynamics, and function using NetworkX,” in Proceedings of the 7th Python in Science Conference (SciPy2008), Pasadena, CA, 11–15

[B18] Hilgetag C. C. Burns G. A. O’Neill M. A. Scannell W. Young M. P. (2000). Anatomical connectivity defines the organization of clusters of cortical areas in the macaque monkey and the cat. Philos. Trans. R. Soc. Lond. B Biol. Sci. 355, 91–110 10.1098/rstb.2000.0551 10703046 PMC1692723

[B19] Honey C. J. Kötter R. Breakspear M. Sporns O. (2007). Network structure of cerebral cortex shapes functional connectivity on multiple time scales. Proc. Natl. Acad. Sci. U.S.A. 104, 10240–10245 10.1073/pnas.0701519104 17548818 PMC1891224

[B20] Honey C. J. Sporns O. Cammoun L. Gigandet X. Thiran J. P. Meuli R. Hagmann P. (2009). Predicting human resting-state functional connectivity from structural connectivity. Proc. Natl. Acad. Sci. U.S.A. 106, 2035–2040 10.1073/pnas.0811168106 19188601 PMC2634800

[B21] Hütt M. T. Jain M. Hilgetag C. C. Lesne A. (2012). Stochastic Resonance in Discrete Excitable Dynamics on Graphs. Chaos: Solitons and Fractals. (in press).

[B22] Hütt M. T. Lesne A. (2009). Interplay between topology and dynamics in excitation patterns on hierarchical graphs. Front. Neuroinform. 3:28 10.3389/neuro.11.028.2009 19826610 PMC2759346

[B23] Kaiser M. Görner M. Hilgetag C. C. (2007a). Criticality of spreading dynamics in hierarchical cluster networks without inhibition. New J. Phys. 9, 110 10.1088/1367-2630/9/5/110

[B24] Kaiser M. Martin R. Andras P. Young M. P. (2007b). Simulation of robustness against lesions of cortical networks. Eur. J. Neurosci. 25, 3185–3192 10.1111/j.1460-9568.2007.05574.x 17561832

[B25] Kaiser M. Hilgetag C. C. (2004). Edge vulnerability in neural and metabolic networks. Biol. Cybern. 90, 311–317 10.1007/s00422-004-0479-1 15221391

[B26] Kaiser M. Hilgetag C. C. (2010). Optimal hierarchical modular topologies for producing limited sustained activation of neural networks. Front. Neuroinform. 4:8 10.3389/fninf.2010.00008 20514144 PMC2876872

[B27] Kauffman S. A. (1969). Metabolic stability and epigenesis in randomly constructed genetic nets. J. Theor. Biol. 22, 437–467 10.1016/0022-5193(69)90015-0 5803332

[B28] Knight R. T. (2007). Neuroscience – neural networks debunk phrenology. Science 316, 1578–1579 10.1126/science.1144677 17569852

[B29] Lewis C. M. Baldassarre A. Committeri G. Romani G. L. Corbetta M. Raichle M. E. (2009). Learning sculpts the spontaneous activity of the resting human brain. Proc. Natl. Acad. Sci. U.S.A. 106, 17558–17563 10.1073/pnas.0905999106 19805061 PMC2762683

[B30] Liao X. H. Qian Y. Mi Y. Xia Q. Z. Huang X. Q. Hu G. (2011). Oscillation sources and wave propagation paths in complex networks consisting of excitable nodes. Front. Phys. 6, 124–132 10.1007/s11467-010-0152-1

[B31] Marr C. Hütt M. T. (2005). Topology regulates pattern formation capacity of binary cellular automata on graphs. Physica A 354, 641–662 10.1016/j.physa.2005.02.019

[B32] Marr C. Hütt M. T. (2006). Similar impact of topological and dynamic noise on complex patterns. Phys. Lett. A 349, 302–305 10.1016/j.physleta.2005.08.096

[B33] Marr C. Hütt M. T. (2009). Outer-totalistic cellular automata on graphs. Phys. Lett. A 373, 546–549 10.1016/j.physleta.2008.12.013

[B34] McGraw P. Menzinger M. (2011). Self-sustaining oscillations in complex networks of excitable elements. Phys. Rev. E 83, 037102 10.1103/PhysRevE.83.037102 21517628

[B35] Milo R. Itzkovitz S. Kashtan N. Levitt R. Shen-Orr S. Ayzenshtat I. Sheffer M. Alon U. (2004). Superfamilies of evolved and designed networks. Science 303, 1538–1542 10.1126/science.1089167 15001784

[B36] Milo R. Shen-Orr S. Itzkovitz S. Kashtan N. Chklovskii D. Alon U. (2002). Network motifs: simple building blocks of complex networks. Science 298, 824–827 10.1126/science.298.5594.824 12399590

[B37] Moreira A. A. Mathur A. Diermeier D. Amaral L. A. N. (2004). Efficient system-wide coordination in noisy environments. Proc. Natl. Acad. Sci. U.S.A. 101, 12085–12090 10.1073/pnas.0400672101 15297617 PMC514439

[B38] Mountcastle V. (1997). The columnar organization of the neocortex. Brain 120, 701–722 10.1093/brain/120.4.701 9153131

[B39] Müller-Linow M. Hilgetag C. C. Hütt M. T. (2008). Organization of excitable dynamics in hierarchical biological networks. PLoS Comput. Biol. 4, e1000190 10.1371/journal.pcbi.1000190 18818769 PMC2542420

[B40] Müller-Linow M. Marr C. Hütt M. T. (2006). Topology regulates the distribution pattern of excitations in excitable dynamics on graphs. Phys. Rev. E 74, 016112 10.1103/PhysRevE.74.016112 16907156

[B41] Penfield W. Jasper P. (1954). Epilepsy and the Functional Anatomy of the Human Brain. London: Little Brown and Company

[B42] Qian Y. Liao X. Huang X. Mi Y. Zhang L. Hu G. (2010a). Diverse self-sustained oscillatory patterns and their mechanisms in excitable small-world networks. Phys. Rev. E 82, 026107 10.1103/PhysRevE.82.026107 20866876

[B43] Qian Y. Huang X. Hu G. Liao X. (2010b). Structure and control of self-sustained target waves in excitable small-world networks. Phys. Rev. E 81, 036101 10.1103/PhysRevE.81.036101 20365809

[B44] Robinson P. A. Henderson J. A. Matar E. Riley P. Gray R. T. (2009). Dynamical reconnection and stability constraints on cortical network architecture. Phys. Rev. Lett. 102, 108104 10.1103/PhysRevLett.103.108104 19792345

[B45] Roxin A. Riecke H. Solla S. A. (2004). Self-sustained activity in a small-world network of excitable neurons. Phys. Rev. Lett. 92, 198101 10.1103/PhysRevLett.92.198101 15169447

[B46] Rubinov M. Sporns O. (2010). Complex network measures of brain connectivity: uses and interpretations. Neuroimage 52, 1059–1069 10.1016/j.neuroimage.2009.10.003 19819337

[B47] Scannell J. W. Burns G. A. Hilgetag C. C. O’Neil M. A. Young M. P. (1999). The connectional organization of the cortico-thalamic system of the cat. Cereb. Cortex 9, 277–299 10.1093/cercor/9.3.277 10355908

[B48] Song S. Sjöström P. J. Reigl M. Nelson S. Chklovskii D. B. (2005). Highly nonrandom features of synaptic connectivity in local cortical circuits. PLoS Biol. 3, e68 10.1371/journal.pbio.0030068 15737062 PMC1054880

[B49] Sporns O. Honey C. Kötter R. (2007). Identification and classification of hubs in brain networks. PLoS ONE 2, e1049 10.1371/journal.pone.0001049 17940613 PMC2013941

[B50] Sporns O. Kötter R. (2004). Motifs in brain networks. PLoS Biol. 2, e369 10.1371/journal.pbio.0020369 15510229 PMC524253

[B51] Sporns O. Tononi G. Edelman G. M. (2000). Theoretical neuroanatomy: relating anatomical and functional connectivity in graphs and cortical connection matrices. Cereb. Cortex 10, 127–141 10.1093/cercor/10.2.127 10667981

[B52] Traub R. D. Schmitz D. Jefferys J. G. R. Draguhn A. (1999). High-frequency population oscillations are predicted to occur in hippocampal pyramidal neuronal networks interconnected by axoaxonal gap junctions. Neuroscience 92, 407–426 10.1016/S0306-4522(98)00755-6 10408594

[B53] van den Heuvel M. P. Mandl R. C. Kahn R. S. Hulshoff Pol H. E. (2009). Functionally linked resting-state networks reflect the underlying structural connectivity architecture of the human brain. Hum. Brain Mapp. 30, 3127–3141 10.1002/hbm.20737 19235882 PMC6870902

[B54] van Vreeswijk C. Sompolinsky H. (1996). Chaos in neuronal networks with balanced excitatory and inhibitory activity. Science 274, 1724–1726 10.1126/science.274.5293.1724 8939866

[B55] van Vreeswijk C. Sompolinsky H. (1998). Chaotic balanced state in a model of cortical circuits. Neural. Comput. 10, 1321–1371 10.1162/089976698300017214 9698348

[B56] Vladimirov N. Tu Y. Traub R. D. (2012). Shortest loops are pacemakers in random networks of electrically coupled axons. Front. Comput. Neurosci. 6:17 10.3389/fncom.2012.00017 22514532 PMC3324298

[B57] Vogels T. P. Abbott L. F. (2005). Signal propagation and logic gating in networks of integrate-and-fire neurons. J. Neurosci. 25, 10786–10795 10.1523/JNEUROSCI.3508-05.2005 16291952 PMC6725859

[B58] Wang S. J. Hilgetag C. C. Zhou C. (2011). Sustained activity in hierarchical modular neural networks: self-organized criticality and oscillations. Front. Comput. Neurosci. 5:30 10.3389/fncom.2011.00030 21852971 PMC3151620

[B59] Wolfram S. (1983). Statistical mechanics of cellular automata. Rev. Mod. Phys. 55, 601–644 10.1103/RevModPhys.55.601

[B60] Wolfram S. (1984). Universality and complexity in cellular automata. Physica D 10, 1–35 10.1016/0167-2789(84)90245-8

[B61] Zamora-López G. Zhou C. Kurths J. (2010). Cortical hubs form a module for multisensory integration on top of the hierarchy of cortical networks. Front. Neuroinform. 4:1 10.3389/neuro.11.001.2010 20428515 PMC2859882

[B62] Zhou C. Zemanová L. Zamora G. Hilgetag C. C. Kurths J. (2006). Hierarchical organization unveiled by functional connectivity in complex brain networks. Phys. Rev. Lett. 97, 238103 10.1103/PhysRevLett.97.155704 17280251

[B63] Zhou C. Zemanová L. Zamora-López G. Hilgetag C. C. Kurths J. (2007). Structure-function relationship in complex brain networks expressed by hierarchical synchronization. New J. Phys. 9, 178 10.1088/1367-2630/9/6/173

